# Performance analysis and optimization design of variant CR-CR 9-speed automatic transmission

**DOI:** 10.1038/s41598-025-93735-6

**Published:** 2025-03-18

**Authors:** Liangyi Nie, Yuqing Meng, Kwun-lon Ting

**Affiliations:** 1 School of Mechanical and Electrical Engineering, Hubei Polytechnic University, Huangshi, 435003 China; 2 Hubei Key Laboratory of Intelligent Convey Technology and Device, Huangshi, 435003 China; 3 School of Mechanical Engineering, Hebei University of Science and Technology, Shijiazhuang, 050018 China; 4 Center for Manufacturing Research, Tennessee Technological University, Cookeville, TN 38505 USA

**Keywords:** Configuration design, Scale synthesis, Automatic transmission, Performance analysis, Optimal design, Mechanical engineering, Mathematics and computing

## Abstract

With the growing demand of people for fuel economy, driving comfort and environmental friendliness of automobiles, the development of high-gear automatic transmissions (ATs) with outstanding performance has become a research focus in the automotive field. However, the lack of systematic research methods has impeded the progress in this field. This paper presents a performance analysis and optimization method for high-gear variant CR-CR 9-speed AT. Firstly, the lever method was employed to calculate the transmission ratios of each gear position, the relative rotational speeds of each component, and the internal and external torques of the transmission, and a general formula for transmission efficiency was derived. Secondly, in order to enhance the performance and efficiency of the transmission, a scaled optimization algorithm was programmed, obtaining the optimal scheme of transmission structure with the highest efficiency and the optimal speed ratio step value ranges for the reduction and acceleration gears. Finally, three-dimensional modeling and simulation were carried out to verify the correctness of the theoretical derivation and the feasibility of the most efficient structure scheme. This method can provide a theoretical foundation and technical support for the improvement and preferred application of high-gear ATs.

## Introduction

An automatic transmission (AT) is a variable-speed device that harmonizes the rotational speed and torque of the engine with the traveling speed and torque of the wheels. It is the core component of the AT system and a key to whether the vehicle achieves its best performance during driving. Enhancing the comprehensive performance of the AT can significantly improve the driving experience of the vehicle in various aspects such as fuel economy, power performance, and driving comfort. The performance factors influencing the AT include the number of gears, the transmission ratio range, the transmission efficiency, and the step ratio, etc. Generally speaking, a transmission with a higher number of gears has a broader transmission range, a relatively smaller step ratio, and a smoother transmission. However, an overly high number of gears will lead to a complex transmission structure, higher manufacturing costs, and a reduced transmission efficiency due to excessive transmission stages, and the maintenance will also be relatively difficult. Therefore, exploring higher gear numbers of ATs while ensuring relatively good transmission efficiency and manufacturing and maintenance costs, and designing ATs with excellent performance is currently the research focus in the field of automotive transmissions. A large number of scholars at home and abroad have conducted relevant studies on this^1–18^.

Li et al.^1^ conducted a systematic analysis of the transmission structure of a 5-speed AT using the equivalent lever method and selected an optimal transmission scheme with three parallel planetary gear trains. Yasuo et al.^2^ established a new shift control system by applying modern control theory, improving the acceleration performance of the 5-speed transmission in the low and medium speed ranges. Zou^3^ established a virtual prototype model of a 6-speed three-row planetary gear mechanism of the AT in Romax software and analyzed the gear safety factor and gear meshing misalignment under various gear conditions, providing a guidance for the design of multi-row complex planetary gear mechanisms. Zhang^4^ analyzed the transmission ratio, size and process parameters, number of working elements and shifting elements of the AT, and proposed a Lepelletier 6-speed AT with the largest transmission ratio range, the smallest fluctuation in speed ratio step change, and fewer working elements and shifting elements. Mercedes-Benz Company^5^ and Daimler-Chrysler Company^6^ launched their respective 7-speed transmissions in 2000. Sun et al.^7^ studied the entire process of die-casting optimization, mold structure optimization, heat treatment, surface treatment and numerical manufacturing of the 7-speed AT housing. Liu et al.^8^ proposed a control strategy for the three stages of rapid oil filling, torque phase and inertia phase based on PI slip control and engine coordination control theory, achieving the engineering application of the 8-speed AT. Wang et al.^9^ investigated the influence of the system pressure and flow control valve on the performance of the 8-speed AT by combining theoretical calculation models and dynamic simulation models. Zhan et al.^10^ analyzed the transmission ratio of the 8-speed AT of ZF Company using the lever method. Traditional 5-, 6-, 7- and 8-speed ATs have undergone a long period of technical precipitation, and their research results are relatively comprehensive and the technology is relatively mature. However, for 9-speed or even higher gear ATs, because they were proposed relatively late, the research on them is relatively scarce and simplistic. Based on the principle of the lever method, Xue et al.^11^ generated transmission schemes that meet the requirements of 9-speed ATs using the exhaustive method. Yang^12^, Cui^13^, Ding et al.^14,15^ synthesized various new configurations of 9-speed ATs using topological graph theory and adjacency matrices. He et al.^16^, Gong et al.^17^, Wang et al.^18^ established the equivalent lever diagrams of each gear of 9- and 10-speed ATs and calculated their transmission ratios.

From the above-mentioned literature, it can be seen that the current research on ATs above high number-speed (such as 9-speed, 10-speed) mainly focuses on the configuration development and transmission ratio calculation of ATs, and there is a lack of more in-depth research on their performance, which will inevitably reduce their performance in use and thus delay their industrial application process. Especially, in the current situation where fuel resources are increasingly tense, the popularization and use of high-gear ATs with excellent performance can undoubtedly improve the fuel economy and driving comfort of automobiles and be more environmentally friendly. Therefore, further research on the performance of high number-speed ATs is extremely urgent and has great application value.

Based on the previous published paper of our team^14,15^, firstly, this paper presents a new configuration variant of the CR-CR 9-speed AT. Secondly, based on the general formulas for kinematic and dynamic analysis derived from the lever method, a scale optimization algorithm is proposed. Then, the range of structural scale values of the AT with high transmission efficiency and smooth speed ratio step values is obtained. After that, a three-dimensional model is established and motion simulation is conducted to prove the feasibility of the proposed method. To sum up, on the one hand, this paper proposes a comprehensive method for performance analysis, which can effectively guide the structural scale design of the 9-speed AT. On the other hand, it can also provide a new type of 9-speed AT configuration, contributing to the advancement of AT research and development and its popularization and application in engineering.

## Variant CR-CR 9-speed automatic transmission

**Fig. 1:**
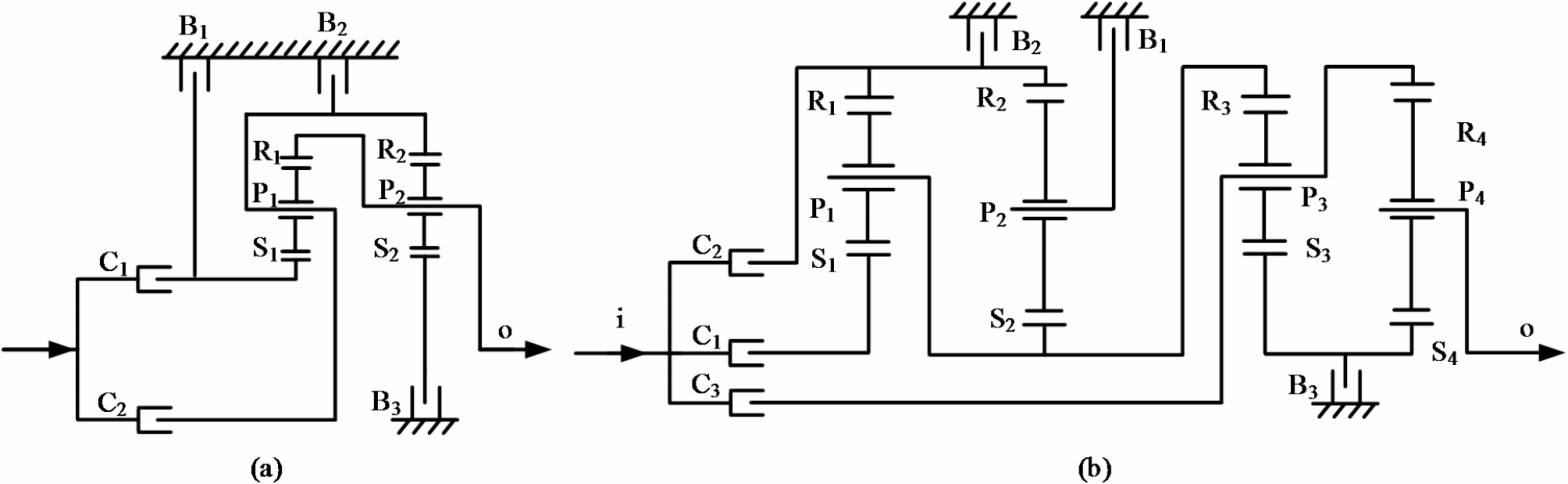
(**a**) A typical CR-CR transmission scheme (**b**) Transmission scheme of the 9-speed automatic transmission.

Traditional high-gear transmissions typically employ two types of planetary gear mechanisms: the Navgneaus type and the CR-CR type^17,23,26,27^. A typical CR-CR transmission scheme^23^ is shown in Fig. 1(a), which consists of two single planetary rows. The sun gears S_1_ and S_2_ of the two planetary rows are independent of each other, and the first planet carrier P_1_ is connected to the second ring gear R_2_, and the first ring gear R_1_ is connected to the second planet carrier P_2_. By arranging different shift actuator elements, up to four forward gears and one reverse gear can be achieved. It has the advantage of a compact structure, but the disadvantage is that there are fewer gear positions. The transmission scheme of the variant CR-CR 9-speed AT is a configurational variation based on the commonly used CR-CR type. It adopts more gear positions (i.e., 9-speed transmission) and by combining fewer control elements, it can achieve a more compact structure, smaller size, greater motion stability, smoother shifting, and provide a more comfortable driving experience. Additionally, this transmission scheme simplifies control and is capable of offering a larger transmission ratio range, enabling lower engine speeds and higher vehicle speeds to meet the power demands under different driving conditions. Furthermore, the variant CR-CR 9-speed AT has undergone innovative configurational design based on the lever method^19,20^, maximizing energy transmission efficiency, minimizing energy loss, enhancing the fuel economy of the vehicle, and extending the service life of the AT.

## Establishment of transmission scheme

The variant CR-CR 9-speed AT is mainly composed of an input shaft, an output shaft, a planetary gear row group, connection components, a torque transfer group, and a transmission housing. Note that, the planetary gear row group adopts a planetary gear train transmission with four planetary gears. At the same time, a new structural form of connecting the inner components of the first three planetary rows (i.e., the first planetary carrier P_1_, the second sun gear S_2_, and the third ring gear R_3_) as a whole is adopted. And the control of each gear only requires the combination of three control elements among the six control elements (Clutch C_1_, C_2_, C_3_, and Brake B_1_, B_2_, B_3_) to achieve the transmission of different gears^21,22^. The transmission schematic diagram of the variant CR-CR 9-speed AT is shown in Fig. 1(b).

**Fig. 2:**
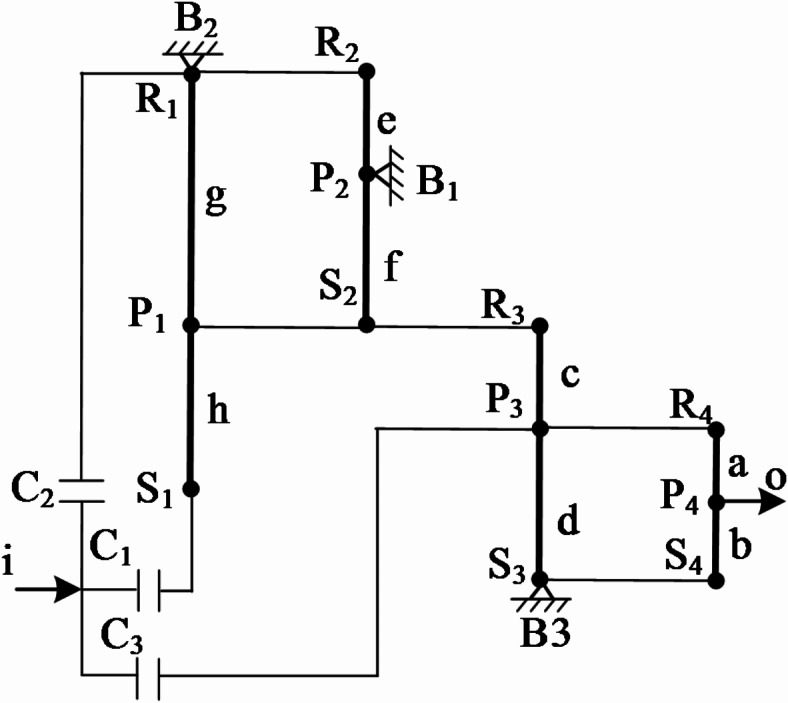
Lever graph of the 9-speed automatic transmission.

In order to better analyze the performance of the variant CR-CR 9-speed AT, it can be transformed into the lever graph as shown in Fig. 2 to explain its working principle more intuitively. It should be noted that Fig. 2 is not the sole form of expression. In principle, as long as the connection relationships of the levers and the layout positions of the coupling elements remain the same, other different forms can be adopted for drawing.

## State of control element

The variant CR-CR 9-speed AT synthesizes gears through distinct input points, output points, and fixed points to form different motion states and achieve various transmission ratios. It thereby possesses nine forward gears and one reverse gear. Among the forward gears, there are four reduction gears, one direct gear, and four acceleration gears, as indicated in Table 1. The solid circle symbol ● here indicates that the control element is in a connected state in this gear position, otherwise it is in a disconnected state.

**Table 1:** Join status of each gear.

Gears	Reduction gears				Direct gear	Acceleration gears				Reverse gear
	1	2	3	4	5	6	7	8	9	10
Control element										
Clutch										
C_1_	●	●	●	●	●	●	●			
C_2_			●		●				●	●
C_3_				●	●	●	●	●	●	
Brake										
B_1_	●						●	●	●	●
B_2_		●				●		●		
B_3_	●	●	●	●						●

## Equivalent lever diagrams for each gear position

**Fig. 3:**
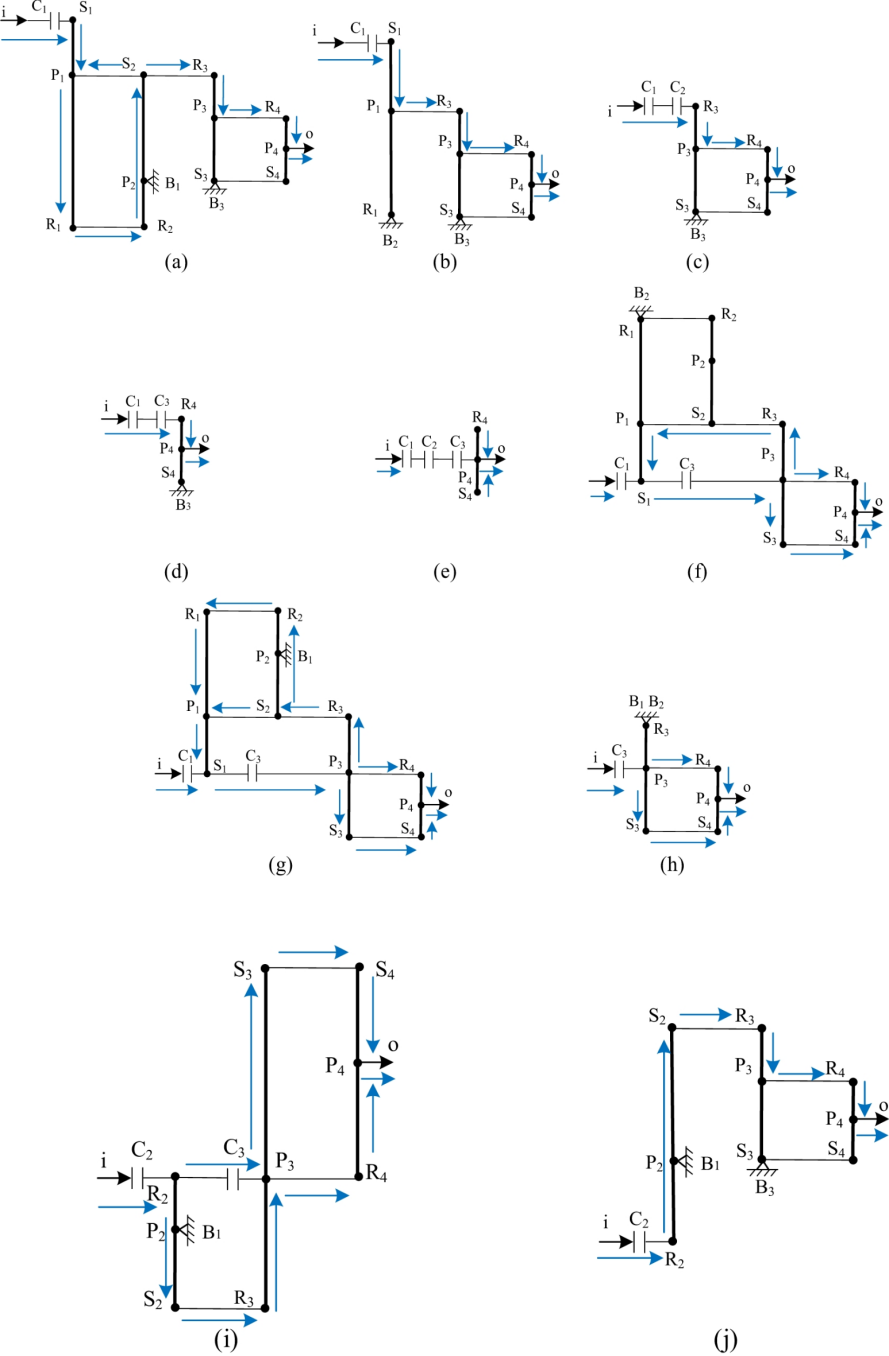
Equivalent lever diagrams for each gear position (**a**) Reduction gear 1, (**b**) Reduction gear 2, (**c**) Reduction gear 3, (**d**) Reduction gear 4, (**e**) Direct gear, (**f**) Acceleration gear 6, (**g**) Acceleration gear 7, (**h**) Acceleration gear 8, (**i**) Acceleration gear 9, (**j**) Reverse gear and corresponding power flow diagrams.

For the variant CR-CR 9-speed AT (Fig. 1b), although only the engagement and disengagement relationship of a pair of control elements need to be changed when shifting between adjacent gears, and the control is relatively simple, the motion transmission process in its transmission scheme diagram is still not clear enough. To solve this problem, it is necessary to draw the transmission lever graphs for each gear position, which can clearly show the planetary gear train motion and transmission for each gear position, as shown in Fig. 3. Noted that only the planet structures that are effective in transmission for that gear position are shown in the equivalent lever diagrams for each gear position, ignoring the planet structures that do not function. This can provide a clearer understanding of the motion process of each planetary gear train in transmission. Additionally, in accordance with the requirements of the reviewers and to enable readers to have a clearer understanding of the working conditions of each gear, based on the research software and data published by our team^22^, we have added the power flow diagrams of each gear in Fig. 3 (i.e., these blue arrows). Among them, there may be power backflow in some gears (such as gear 1, gear 6, and gear 7). However, since this paper only involves the research of the transmission scheme of the AT and cannot consider the complex factors such as the working conditions and the shift strategy, the corresponding critical conditions of the gears cannot be set yet. But this will be the research focus of our next step.

## Kinematic analysis

The motion situation of planetary gear train speed transmission is relatively complex, and it is difficult to clearly reflect the transmission performance of the CR-CR 9-speed AT by relying on the schematic diagram and the state table of the control element. Therefore, it is necessary to conduct kinematic analysis to deeply understand its motion characteristics^23^. Studying the motion states and motion relationships of each component of the transmission enables the determination of the transmission ratio and the relative rotational speed formulas of each component and control elements (clutches and brakes), in order to adapt to the transmission ratio change requirements under different driving conditions, providing a significant reference for the subsequent scale optimization design and enhancing the smoothness and efficiency of the AT.

## Establishment of transmission ratios for each gear position

**Fig. 4:**
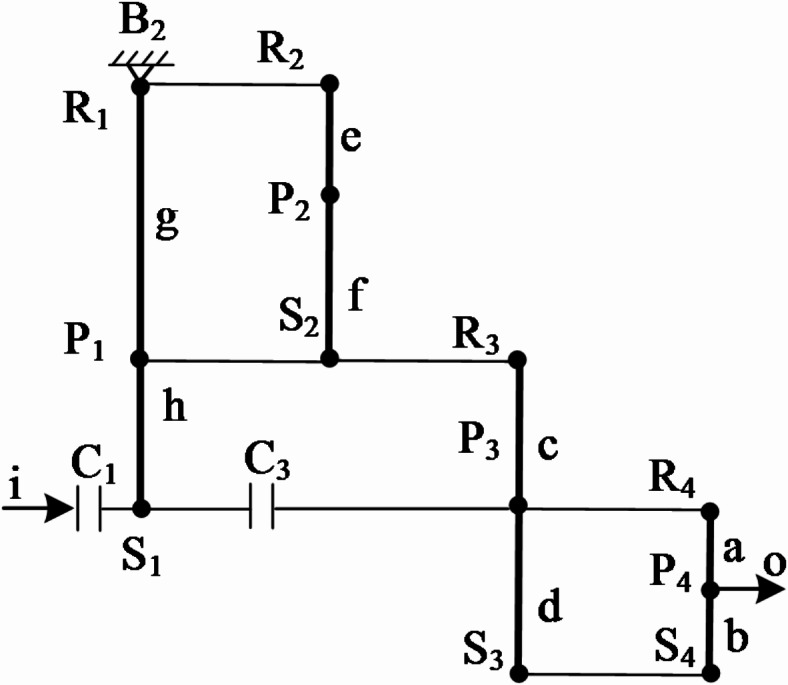
Equivalent lever diagram of the acceleration gear 6.

The transmission ratio of an AT refers to the speed ratio between the input and output components. The magnitude of the transmission ratio represents the rate of speed change of the transmission, and its symbol ± indicates the identity or difference in the rotational direction between the input and the output. To illustrate the calculation method through lever analysis, the acceleration gear 6 of the variant CR-CR 9-speed AT is taken as an example in this paper. The characteristic parameters of planetary gear trains, *K*_*i*_ (*i* = 1, 2, 3, 4), are defined as *K*_*1*_ = *h/g*, *K*_*2*_ = *f/e*, *K*_*3*_ = *d/c* and *K*_*4*_ = *b/a*. These parameters *a*, *b*, *c*, *d*, *e*, *f*, *g*, and *h* correspond to the length of components in the gear train which can be found in Fig. 4; Table 2. For simplicity in calculations, *a* = 1 is used as the standard scale for all other lengths^24,25^, and their values are shown in Table 2.

**Table 2:** The lengths of each segment indicated by K values.

Parameter	Value
*a*	1
*b*	*K*_*4*_
*c*	*(K*_*4*_ *+* 1*)/K*_*3*_
*d*	*K*_*4*_ *+ 1*
*e*	*(K*_*4*_ *+* 1*)/(K*_*2*_ *+* 1*)K*_*1*_*K*_*3*_
*f*	*(K*_*4*_ *+* 1*)K*_*2*_*/(K*_*2*_ *+* 1*)K*_*1*_*K*_*3*_
*g*	*(K*_*4*_ *+* 1*)/K*_*1*_*K*_*3*_
*h*	*(K*_*4*_ *+* 1*)/K*_*3*_

Based on lever method, in the corresponding equivalent lever diagram, the transmission ratio *i* is defined as the ratio of the length *n*_*i*_ from the input position to the braking point position to the length *n*_*o*_ from the output position to the same braking point position, which can be written as Eq. 1.

1 $$i=\frac{{{n_i}}}{{{n_o}}}$$

It can be concluded from Table 2 that the transmission ratio *i*_*6*_ of the acceleration gear 6 is:

2 $${i_6}=\frac{{{n_i}}}{{{n_o}}}=\frac{{g+h}}{{g+h+a}}=\frac{{({K_1}+{K_4})({K_4}+{\text{1}})}}{{{\text{1}}+{K_1}+{K_4}+{K_1}{K_3}+{K_1}{K_4}}}$$

Similarly, other gear transmission ratios can be obtained, as shown in Table 3.

**Table 3:** Transmission ratio of each gear.

Gear	Transmission ratio
1	*(K*_*1*_ *+ K*_*2*_ *+ K*_*1*_*K*_*2*_*)(K*_*4*_ *+* 1*)(K*_*3*_ *+ 1)/K*_*2*_*K*_*3*_*K*_*4*_
2	*(K*_*1*_ *+* 1*)(K*_*3*_ *+* 1*)(K*_*4*_ *+* 1*)/K*_*3*_*K*_*4*_
3	*(K*_*3*_ *+* 1*)(K*_*4*_ *+* 1*)/K*_*3*_*K*_*4*_
4	*(K*_*4*_ *+* 1*)/K*_*4*_
5	1
6	*(K*_*1*_ *+* 1*) (K*_*4*_ *+* 1*)/(*1 *+ K*_*1*_ *+ K*_*4*_*+ K*_*1*_*K*_*3*_*+ K*_*1*_*K*_*4*_*)*
7	*(K*_*1*_ *+ K*_*2*_ *+ K*_*1*_*K*_*2*_*)(K*_*4*_ *+* 1*)/[K*_*1*_*K*_*3*_*(K*_*2*_ *+* 1*)+(K*_*1*_ *+ K*_*2*_ *+ K*_*1*_*K*_*2*_*)(K*_*4*_ *+* 1*)]*
8	*(K*_*4*_ *+* 1*)/(*1 *+ K*_*3*_ *+ K*_*4*_*)*
9	*(K*_*4*_ *+* 1*)/(*1 *+ K*_*2*_*K*_*3*_ *+ K*_*3*_ *+ K*_*4*_*)*
10	*− (K*_*3*_ *+* 1*)(K*_*4*_ *+* 1*)/K*_*2*_*K*_*3*_*K*_*4*_

## Rotational speed analysis of each gear

In Fig. 4, the equivalent lever diagram of the acceleration gear 6 shows that the transmission motion at this gear position is obtained through the cooperation of brake B_2_ and clutches C_1_, C_3_. Since brake B_2_ is combined with the first and second ring gears and cannot rotate, the motion of the input shaft is transmitted through clutches C_1_ and C_3_ successively to the first sun gear S_1_, the third planet carrier P_3_, the fourth ring gear R_4_ and the planet carrier P_4_, thereby achieving an acceleration of the output speed. To better analyze the rotational speeds of the gear components, the subsequent process constructs the corresponding rotational speed diagram through a series of three defined steps.

**Fig. 5:**
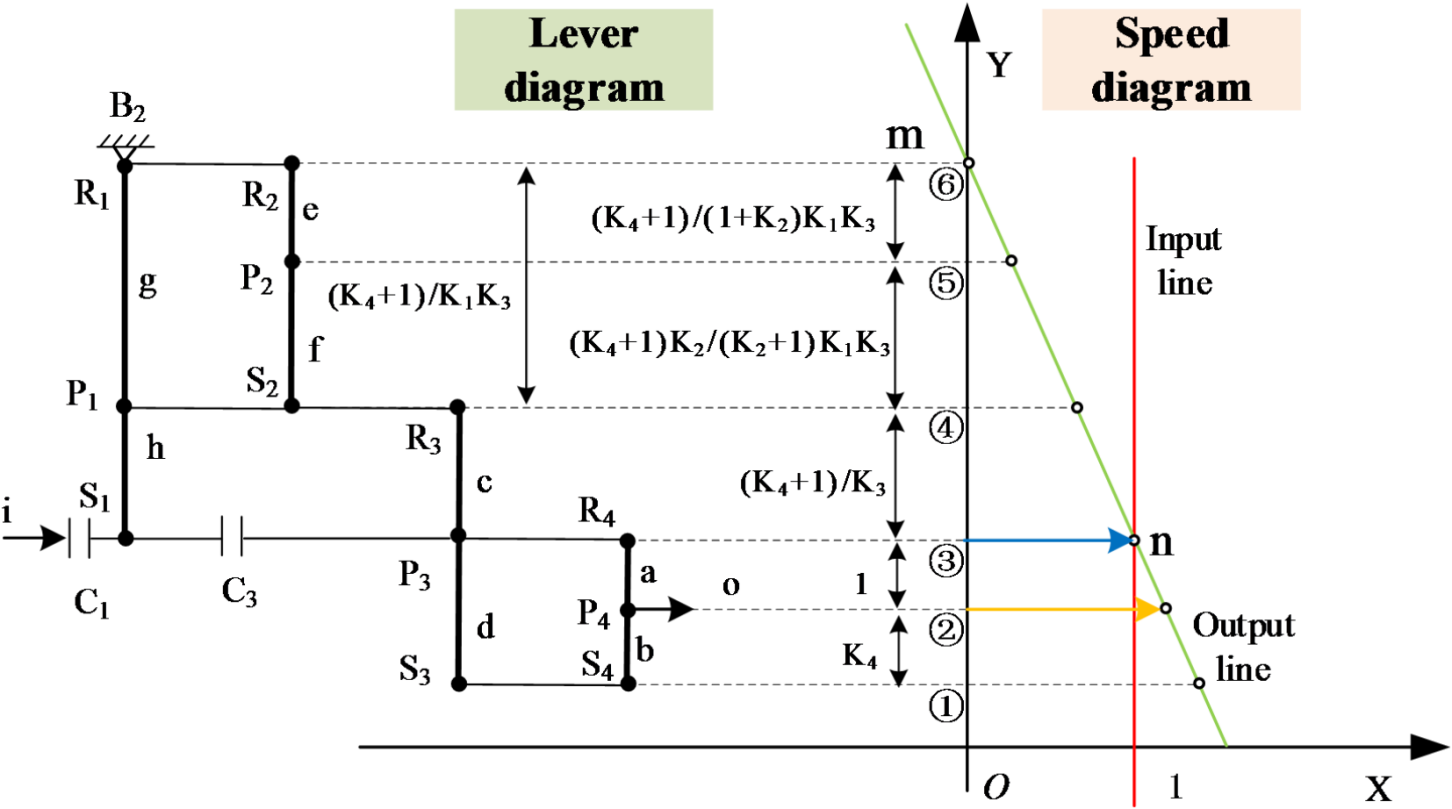
Rotational speed diagram of the acceleration gear 6.

*Step 1 : Establish a coordinate system*. As shown in Fig. 5, a rectangular coordinate system XOY are established. Here, O is the intersection point of the X-axis and the Y-axis, and its coordinate value is (0, 0). The values on the X-axis represent the magnitude of the rotational speed, and the direction with the arrow is the positive direction^26^. On the left side of the Y-axis is the lever diagram, and on the right side of the Y-axis is the corresponding speed diagram. And for speed diagram, the Y-axis is the reference line for positive and negative velocities. Besides this, it is worth noting that the vertical axis of the lever diagram will be parallel to the Y-axis, and the input direction of the lever diagram is parallel to the X-axis.

*Step 2 : Equivalently convert the lever diagram*. Under the above-mentioned coordinate system, the planetary gear transmission mechanism of this gear position (i.e., the lever diagram shown in Fig. 5) is further simplified and can be equivalent to a 6-point lever composed of points ①, ②, ③, ④, ⑤, ⑥. Among them, ①, ②, ③, ④, ⑤, ⑥ represent different components in the transmission mechanism (① represents S_3_ and S_4_, ② represents P_4_, ③ represents S_1_, P_3_, and R_4_, ④ represents P_1_, S_2_, and R_3_, ⑤ represents P_2_, and ⑥ represents R_1_ and R_2_), formed by the intersection of the extended horizontal axis of the lever diagram with the Y-axis of the coordinate system.

*Step 3 : Build speed diagram*. For the sake of calculation convenience, it is assumed that the input speed of the input is + 1, which is represented as a straight line passing through the coordinate (1, 0) and parallel to the Y-axis in Fig. 5, that is, the red input line. Through analysis, the coordinate of point ***m*** is (0, y_⑥_), which mean that its rotational speed is 0. Therefore, its length in the speed diagram is 0, indicating that gear rings R_1_ and R_2_ are connected to the fixed base. ***n*** is the intersection point of the input line of the lever diagram passing through point ③ and the input line of the speed diagram, and its coordinate is (1, y_③_). The blue line segment with an arrow in the speed diagram is its velocity magnitude, that is, the velocity is 1. The straight green line obtained by connecting points ***m*** and ***n*** is the output line of the speed diagram. By extending the horizontal axis of the lever diagram through the corresponding points to form intersections with the output lines of the speed diagram (i.e., the hollow circle symbol ○), the velocity information of each point can be obtained. For example, the velocity of point ② is the yellow arrowed line segment. The length of the velocity line segment can be obtained by the method of similar triangles with the help of the *K*_*i*_-value information of the lever diagram. The length of the velocity line segment of point ② is *((K*_*1*_ *+ 1)(K*_*4*_ *+ 1) + K*_*1*_*K*_*3*_*)/(K*_*1*_ *+ 1)(K*_*4*_ *+ 1).* If the magnitude of the calculated speed line segment is negative, it indicates that its rotational speed direction is opposite to the positive direction of the X-axis, and it is used as the reverse gear.

## Relative rotational speed of control element

Since the control elements, namely clutches and brakes, are the fundamental connection components used to link two components in different planetary gearsets, their relative rotational speeds can be computed based on the difference between the rotational speeds of the two connected components. When the transmission is in the acceleration gear 6, brake B_2_ is engaged and connected to the transmission casing, thus their rotational speeds are zero. When B_2_ is in the disengaged state, the relative rotational speeds of brakes B_1_ and B_3_ are equivalent to the rotational speeds of the connected components. When clutches C_1_ and C_3_ are in operation, since the rotational speeds of the two components coupled by C_1_ and C_3_ are identical, the relative rotational speed of the clutches in the working state is 0. And the rotational speed of clutch C_2_ in the disengaged state can be derived from the difference between the rotational speeds of the two components, its value is shown in Table 4. For the transmission, through the coordinated use of clutches and brakes, the components between different planetary gearsets can be connected, and the rationality of their relative rotational speeds can be ensured.

**Table 4:** Relative rotational speed of control element.

Control element	Relative rotational speed
Brake	
B_1_	1/(1 + *K*_*1*_*K*_*3*_)
B_2_	0
B_3_	*K*_*1*_*K*_*3*_/(1 *+ K*_*1*_*K*_*3*_)
Clutch	
C_1_	0
C_2_	1
C_3_	0

### Torque analysis

To further examine the feasibility of the designed transmission mechanism and transmission scheme, a dynamic performance analysis of the planetary gear transmission mechanism based on the lever method is necessary. This involves investigating the transfer of forces and moments among the various components of the transmission, as well as the mechanical and dynamic equilibria of the system, to endow the transmission with better acceleration and adaptability. In the following part, still taking the acceleration gear 6 of the AT as an example, the solution process for the internal and external torques and the transmission efficiency endured by each component in the mechanism is mainly elaborated^27^.

### External torque of planetary gear transmission mechanism

**Fig. 6:**
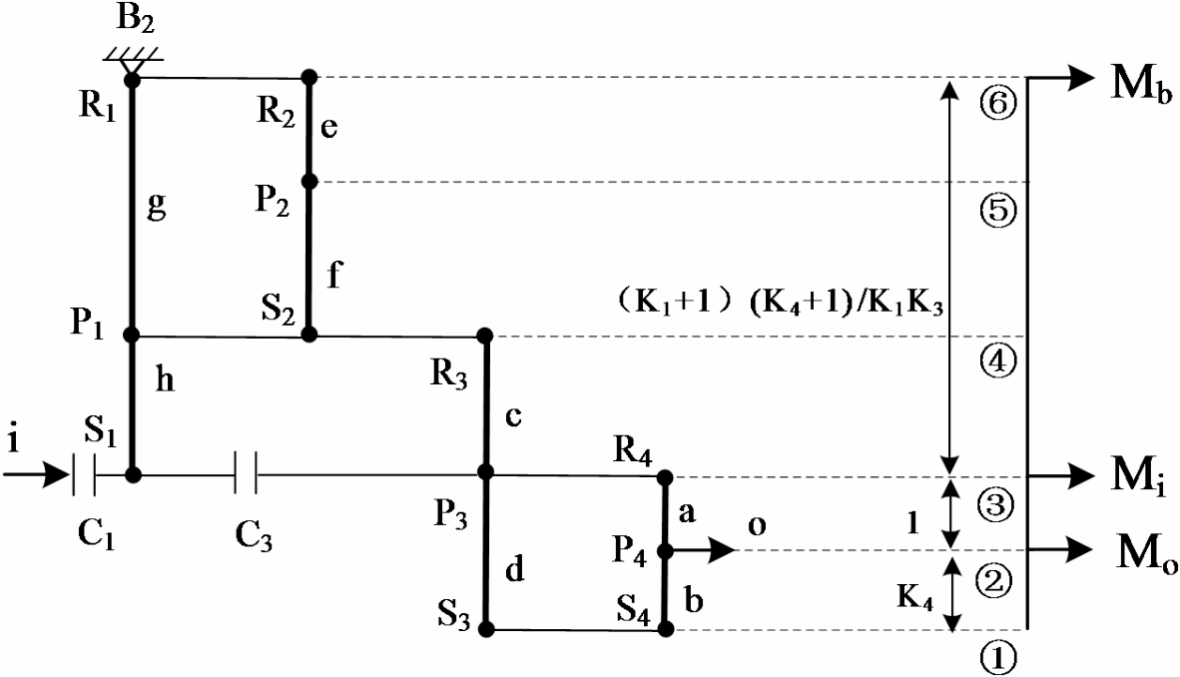
Torque analysis diagram of the acceleration gear 6.

Assuming that the input torque *M*_*i*_ is known, the action point is at point ③; the output torque *M*_*o*_ acts at point ②; and the driving torque *M*_*b*_ acts at point ⑥, as shown in Fig. 6.

Based on the principle of force balance, Eqs. (3) and (4) can be obtained as followed,

3 $${M_i}+{M_o}+{M_b}=0$$

4 $$\left\{ {\begin{array}{*{20}{l}} {{M_{\text{o}}}={M_b}({K_1}+{\text{1}})({K_4}+{\text{1}})/{K_1}{K_3}} \\ {{M_i}= - {M_o} - {M_b}} \end{array}} \right.$$

For the convenience of calculation, make ,$A=\frac{{({K_1}+{\text{1}})({K_4}+{\text{1}})}}{{1+{K_1}+{K_4}+{K_1}{K_3}+{K_1}{K_4}}}$, Eqs. (3) and (4) can be simplified as followed,

5 $$\left\{ {\begin{array}{*{20}{l}} {{M_o}=-A{M_i}} \\ {{M_b}=(A-{\text{1}}){M_i}} \end{array}} \right.$$

## Internal torque of each planetary row

The internal torque of the planetary gear train refers to the torque that enables the meshing of the planetary gears with the sun gear and the ring gear to achieve transmission. For the convenience of calculation, usually, the analysis begins with a component that is subjected to only a single known external torque and does not participate in multi-row transmission. Then, based on the principle of torque balance, the internal torques received by other components are solved step by step. In this paper, the analysis starts from the planet carrier of the fourth planetary gear train that is subjected only to the output torque. The internal torque of its planet is obtained as followed:

6 $${M_{{P_4}}}= - {M_o}=A{M_i}$$

According to the principle of torque balance, the internal torque of the gear ring of the fourth planetary gear train and the sun gear can be calculated as follows,

7 $$\left\{ {\begin{array}{*{20}{l}} {{M_{{R_4}}}={{ - A{K_4}{M_i}} \mathord{\left/ {\vphantom {{ - A{K_4}{M_i}} {({K_4}+1)}}} \right. \kern-0pt} {({K_4}+1)}}} \\ {{M_{{S_4}}}={{ - A{M_i}} \mathord{\left/ {\vphantom {{ - A{M_i}} {({K_4}+1)}}} \right. \kern-0pt} {({K_4}+1)}}} \end{array}} \right.$$

8 $${M_{{S_3}}}= - {M_{{S_4}}}={{A{M_i}} \mathord{\left/ {\vphantom {{A{M_i}} {({K_4}+1)}}} \right. \kern-0pt} {({K_4}+1)}}$$

Similarly, the internal torques of the gear rings of the third, second, and first planetary gear trains and the sun gears, their internal torque can be obtained in turn as followed,

9 $$\left\{ {\begin{array}{*{20}{l}} {{M_{{R_3}}}={{A{K_3}{M_i}} \mathord{\left/ {\vphantom {{A{K_3}{M_i}} {({K_4}+1)}}} \right. \kern-0pt} {({K_4}+1)}}} \\ {{M_{{P_3}}}={{ - A({K_3}+1){M_i}} \mathord{\left/ {\vphantom {{ - A({K_3}+1){M_i}} {({K_4}+1)}}} \right. \kern-0pt} {({K_4}+1)}}} \end{array}} \right.$$

10 $${M_{{S_2}}}= - {M_{{{\text{R}}_3}}}={{ - A{K_3}{M_i}} \mathord{\left/ {\vphantom {{ - A{K_3}{M_i}} {({K_4}+1)}}} \right. \kern-0pt} {({K_4}+1)}}$$

11 $$\left\{ {\begin{array}{*{20}{l}} {{M_{{{\text{R}}_2}}}={{ - A{K_2}{K_3}{M_i}} \mathord{\left/ {\vphantom {{ - A{K_2}{K_3}{M_i}} {({K_4}+1)}}} \right. \kern-0pt} {({K_4}+1)}}} \\ {{M_{{{\text{P}}_2}}}={{A{K_3}(1+{K_2}){M_i}} \mathord{\left/ {\vphantom {{A{K_3}(1+{K_2}){M_i}} {({K_4}+1)}}} \right. \kern-0pt} {({K_4}+1)}}} \end{array}} \right.$$

12 $${M_{{{\text{P}}_1}}}= - {{{M_{{{\text{S}}_2}}}=A{K_3}{M_i}} \mathord{\left/ {\vphantom {{{M_{{{\text{S}}_2}}}=A{K_3}{M_i}} {({K_4}+1)}}} \right. \kern-0pt} {({K_4}+1)}}$$

13 $$\left\{ {\begin{array}{*{20}{l}} {{M_{{R_1}}}={{ - A{K_1}{K_3}{M_i}} \mathord{\left/ {\vphantom {{ - A{K_1}{K_3}{M_i}} {({K_1}+1)({K_4}+1)}}} \right. \kern-0pt} {({K_1}+1)({K_4}+1)}}} \\ {{M_{{S_1}}}={{ - A{K_3}{M_i}} \mathord{\left/ {\vphantom {{ - A{K_3}{M_i}} {({K_1}+1)({K_4}+1)}}} \right. \kern-0pt} {({K_1}+1)({K_4}+1)}}} \end{array}} \right.$$

Since the braking torque is provided by brake B2, it can be calculated as Eqs. (14) and (15).

14 $${M_{{{\text{B}}_2}}}= - {M_{{{\text{R}}_1}}} - {M_{_{{{{\text{R}}_2}}}}}$$

15 $${M_{{{\text{B}}_2}}}=\frac{{A{K_1}{K_3}{M_i}}}{{({K_1}+1)({K_4}+1)}}+\frac{{A{K_2}{K_3}{M_i}}}{{({K_4}+1)}}$$

## Analysis of transmission efficiency

The transmission efficiency is an important indicator of the performance of planetary gear trains, and its value is the ratio of the actual transmission ratio to the theoretical transmission ratio as shown in Eq. (16)^14, 26^.

16 $$\mu ={{\hat {i}} \mathord{\left/ {\vphantom {{\hat {i}} i}} \right. \kern-0pt} i}$$

Where *µ* is the transmission efficiency of planetary gear mechanism; *i* is the theoretical force transmission ratio of the transmission,$i=f({K_1},{K_2}, \cdot \cdot \cdot ,{K_l}, \cdot \cdot \cdot ,{K_n})$; $\hat {i}$is the actual force transmission ratio, $\hat {i}=({K_1}h_{c}^{{{X_1}}},{K_2}h_{c}^{{{X_2}}}, \cdot \cdot \cdot ,{K_l}h_{c}^{{{X_l}}}, \cdot \cdot \cdot ,{K_n}h_{c}^{{{X_n}}})$; *n* is the number of planetary rows in the planetary gear mechanism; *h*_*c*_ is the transmission efficiency of the planetary row when the planetary carrier is fixed. Usually, it is 0.97 for a single planet and 0.95 for a double planet^27^. ${X_n}=sign(\frac{{\partial \ln i}}{{\partial {K_n}}})$, when $\frac{{\partial \ln i}}{{\partial {K_n}}}>0$,${X_n}=+1$, other wise, ${X_n}= - 1$(*n* = 1, 2, 3,., n). Still taking the acceleration gear 6 as an example, the obtained transmission ratio and partial derivative are shown in Table 5. By comparing the four groups of partial derivatives in the acceleration gear 6 with the size of 0, the value of *X*_*n*_ can be obtained, and then all the *K*_*n*_ in *i*_*6*_ can be replaced by ${K_n}h_{c}^{{{X_n}}}$ to obtain ${\hat {i}_6}$, after that the transmission efficiency ${m_6}={{{{{\hat {i}}_6}} \mathord{\left/ {\vphantom {{{{\hat {i}}_6}} i}} \right. \kern-0pt} i}_6}$ can be solved. Similarly, the transmission efficiency of other gears can be acquired.

**Table 5:** Transmission ratio and partial derivative in the acceleration gear 6.

Transmission ratio${i_6}$	$\frac{{{n_i}}}{{{n_o}}}=\frac{{({K_1}+1)({K_4}+1)}}{{1+{K_1}+{K_4}+{K_1}{K_3}+{K_1}{K_4}}}$
$ln{i_6}$	$\ln [({K_1}+1)({K_4}+1)] - \ln (1+{K_1}+{K_4}+{K_1}{K_3}+{K_1}{K_4})$
Partial derivative	
$\frac{{\partial \ln {i_6}}}{{\partial {K_1}}}$	$\frac{{{K_4}+1}}{{({K_1}+1)({K_4}+1)}} - \frac{{{K_3}+{K_4}+1}}{{1+{K_1}+{K_4}+{K_1}{K_3}+{K_1}{K_4}}}$
$\frac{{\partial \ln {i_6}}}{{\partial {K_2}}}$	0
$\frac{{\partial \ln {i_6}}}{{\partial {K_3}}}$	$- \frac{{{K_3}}}{{1+{K_1}+{K_4}+{K_1}{K_3}+{K_1}{K_4}}}$
$\frac{{\partial \ln {i_6}}}{{\partial {K_4}}}$	$\frac{{{K_1}+1}}{{({K_1}+1)({K_4}+1)}} - \frac{{{K_1}+1}}{{1+{K_1}+{K_4}+{K_1}{K_3}+{K_1}{K_4}}}$

## Scale optimization algorithm

Based on the performance analyses of the planetary gear transmission in Sects. 3 and 4, the general formulas for the kinematic and dynamic performance analysis of each gear position can be obtained. These formulas can offer an analytical approach for the performance of the 9-speed AT. However, the specific performance indicators of this transmission remain undetermined due to the lack of determination for the characteristic parameters values of the planetary gear train *K*_*i*_ (*i* = 1, 2, 3, 4), which hinders the design and manufacture of this new type of transmission. As transmission efficiency is a key indicator of the performance of ATs, it is conducive to optimizing the structural parameters of the components and enhancing the overall performance and driving experience of the AT. To address the aforementioned issues, this paper will employ Python to write a scale optimization algorithm for the 9-speed AT, which takes transmission efficiency as the optimal indicator and regulating the values of the characteristic parameters of the planetary gear train as the means.

**Fig. 7:**
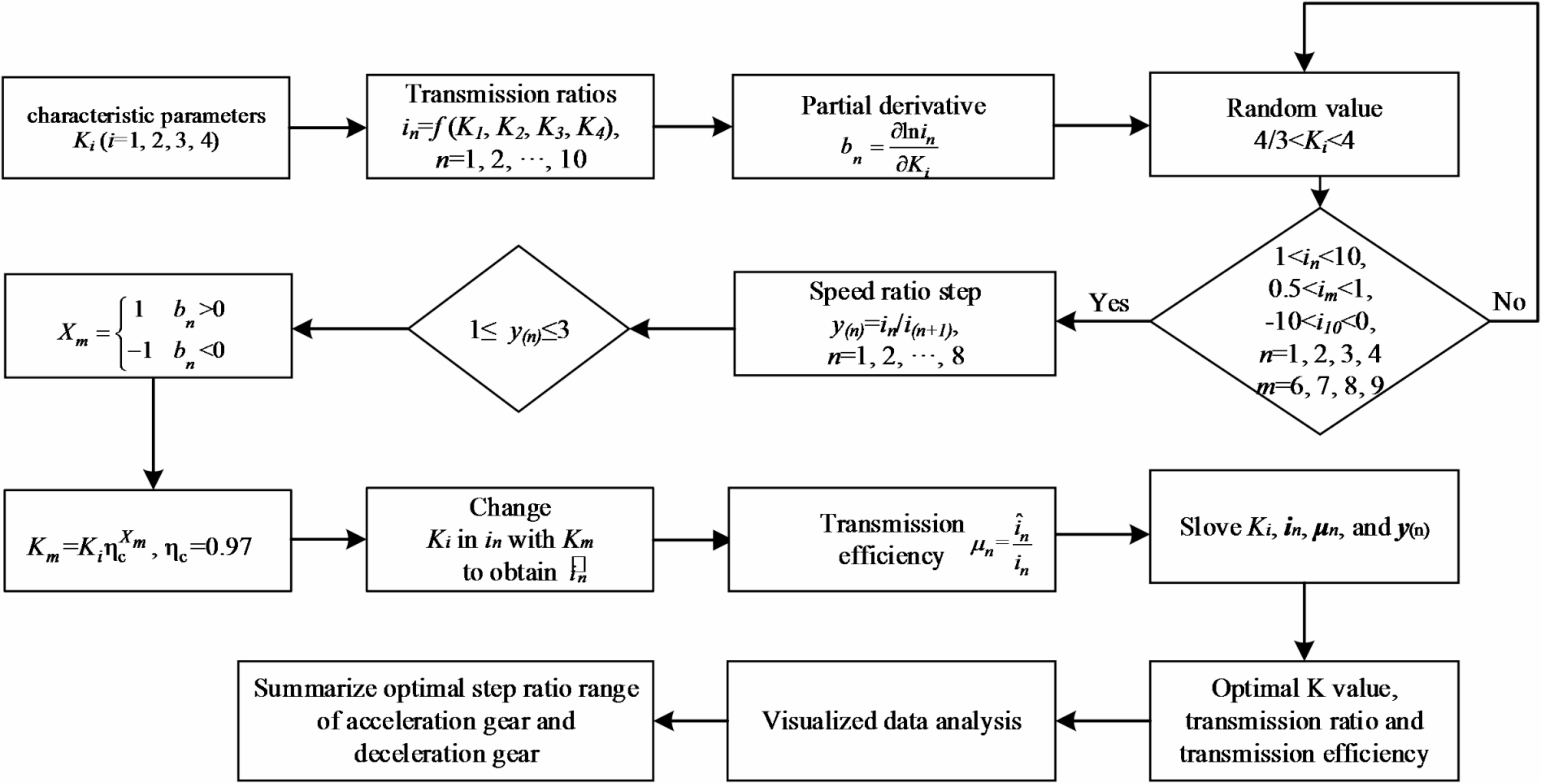
Flow chart of scale optimization algorithm.

Firstly, this algorithm acquires a set of initial data, including the characteristic parameters of the planetary gear train, corresponding transmission ratios, and efficiencies, based on a set of constraints through the enumeration method. For the variant CR-CR 9-speed AT, based on the discussion in Sect. 4.3, it can be concluded that the transmission efficiency of each gear position can be obtained in accordance with the four characteristic parameters of planetary gear trains *K*_*i*_ (*i* = 1, 2, 3,4) and the ten transmission ratios of gear positions *i*_*j*_ (*j* = 1,2, …,10). What’s more, the empirical value range of K_i_ is from 4/3 to 4, the value range of transmission ratio for the reduction gears (*i*_*1*_, *i*_*2*_, *i*_*3*_, *i*_*4*_) is from 1 to 10. For the direct gear, its value of transmission ratio (*i*_*5*_) is 1, and the value range of transmission ratios for the acceleration gear (*i*_*6*_, *i*_*7*_, *i*_*8*_, *i*_*9*_), it is from 0.5 to 1. For the reverse gear, and its value range of transmission ratio (*i*_*10*_) is from − 10 to 0^27^. Therefore, in this algorithm, the initial values of *K*_*i*_ and *i*_*j*_ will be randomly selected within these ranges. Then, with the step value of the speed ratio ranging from 1 to 3 as the preliminary constraint condition, the planet gear characteristic parameters *K*_*i*_ and the transmission ratios *i*_*j*_ that meet the requirements are preliminarily screened. Meanwhile, the actual transmission ratio and transmission efficiency are obtained by using the partial derivative formula in Table 5. Since each set of *K*_*i*_ values will correspond to ten transmission ratios *i*_*j*_, ten transmission efficiencies, and eight speed ratio steps excluding the reverse gear, the optimal *K*_*i*_ value, transmission ratio *i*_*j*_, and the transmission efficiency of each gear position can be screened out based on the average transmission efficiency. And through the assignment of *K*_*i*_ and *i*_*j*_ by enumeration, a large amount of data can be obtained in this algorithm, forming a visual graph. Subsequently, the range of the step ratio is narrowed through comparative analysis of visual data. Finally, through data classification and summarization, the optimized data for the step ratio range applicable to the deceleration gear and the acceleration gear are comprehensively derived. The flowchart of the algorithm is depicted in Fig. 7.

## Example verification

In this part, the aforementioned scale optimization algorithm will be utilized on the variant CR-CR 9-speed AT to validate the correctness and feasibility of the theory. Initially, the algorithm will be applied to acquire a considerable amount of research data (*K*_*i*_, *i*_*j*_, and *m*_*i*_), and subsequently, visualization graphs will be generated for visual data analysis. The parameter range will be further refined until the transmission performance attains the optimal state. Eventually, 3D simulation functional emulation will be conducted.

## Data visualization analysis

### Visualized graph

It is widely acknowledged that, typically, the superiority or inferiority of an AT’s transmission scheme is closely associated with the range of transmission ratios and the speed ratio step. The larger the transmission ratio range and the smaller the speed ratio step, the more gear positions the AT can achieve, and the smoother the gear shifting process will be^28^, leading to better performance. Under the precondition of guaranteeing the same performance requirements, a larger transmission ratio range can be attained through multiple approaches and is relatively easier, while achieving a smaller speed ratio step is more challenging. Nevertheless, for high-gear ATs, the speed ratio step can better reflect the performance quality of the AT. Hence, in this paper, for the variant CR-CR 9-speed AT, based on the proposed algorithm, all the *K*_*i*_ values, transmission ratios, and transmission efficiencies that meet the preferred conditions are screened out. On this basis, through visualized graph of the speed ratio step, the optimal transmission scheme and transmission range are selected. This two steps can be summarized to obtained the visualized graph as follows.

**Fig. 8:**
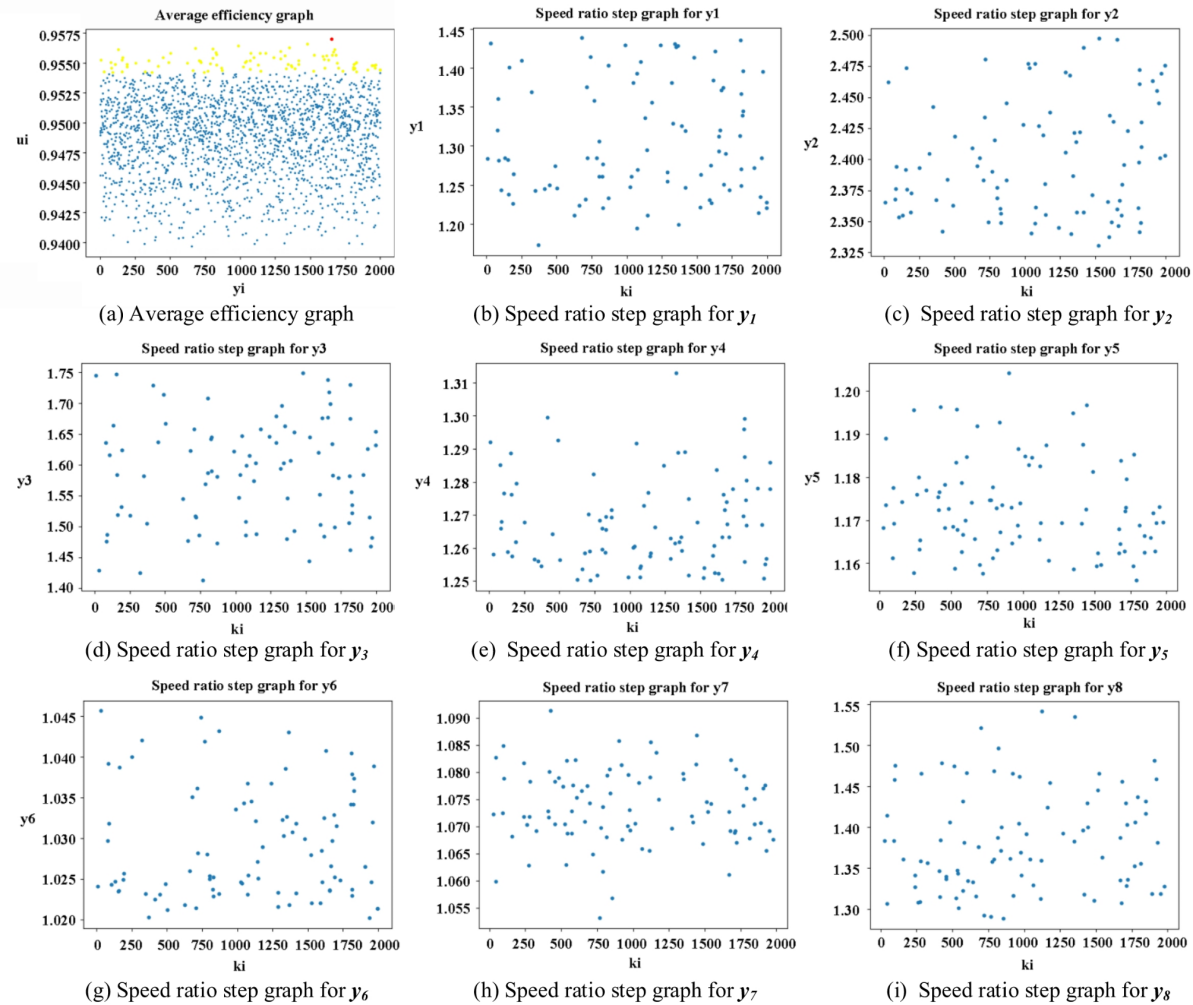
Visualized graph of the relationship between average efficiency and speed ratio step and the *K*_*i*_ value.

Step 1: *Determine the* *K*_*i*_ *values that meet the conditions*,* the corresponding transmission efficiency*,* and the speed ratio step by step through programming*,* and these data are collated into a dataset.* Since each transmission scheme has four *K*_*i*_ values corresponding to 10 transmission efficiencies, the four *K*_*i*_ values of each transmission scheme are represented in the form of group numbers, and the transmission efficiency are taken the average value for simplifying the obtained visualized graph. For example, the four *K*_*i*_ values of the first group are denoted as ***k***_***1***_, and the average transmission efficiency is recorded as ***u***_***1***_; the four *K*_*i*_ values of the second group are denoted as ***k***_***2***_, and the average transmission efficiency is recorded as ***u***_***2***_; the four *K*_*i*_ values of the ***i***-th group are denoted as ***k***_***i***_, and the average transmission efficiency is recorded as ***u***_***i***_. Figure 8(a) is the average efficiency graph. The X-axis of this graph is ***k***_***i***_, and the Y-axis is ***u***_***i***_. Each point in the graph respectively represents a set of *K*_*i*_ values and the corresponding average transmission efficiency.

Step 2: *Based on the* *K*_*i*_ *value*,* the speed ratio steps of the eight gear can be determined*,* forming the speed ratio step graph.* For the variant CR-CR 9-speed AT, the speed ratio step from the first gear to the second gear is recorded as ***y***_***1***_, the speed ratio step from the second gear to the third gear is recorded as ***y***_***2***_, the speed ratio step from the third gear to the fourth gear is recorded as ***y***_***3***_, the speed ratio step from the fourth gear to the fifth gear is recorded as ***y***_***4***_, the speed ratio step from the fifth gear to the sixth gear is recorded as ***y***_***5***_, the speed ratio step from the sixth gear to the seventh gear is recorded as ***y***_***6***_, the speed ratio step from the seventh gear to the eighth gear is recorded as ***y***_***7***_, and the speed ratio step from the eighth gear to the ninth gear is recorded as ***y***_***8***_. In the average efficiency graph obtained in Step 1, several points with average efficiency values approximating 1 are selected. The corresponding *K*_*i*_ values are extracted, and the corresponding speed ratio step values are solved to form the speed ratio step graph such as Fig. 8(b–i). And this graph has ***k***_***i***_ as the x-axis and ***y***_***i***_ as the y-axis.

### Graph analysis

In Fig. 8(a), the yellow and red points represent the average efficiency values that fall within the range of 0.9542 to 1, totaling 91 points, with the red points representing the point with the maximum average efficiency value (0.9572). In general, for high-performance ATs, the closer the average efficiency value is to 1, the smaller the speed ratio step is, indicating better performance. Based on the corresponding *K*_*i*_ values and speed ratio steps for the 91 points, the speed ratio step graphs are formed as shown in Fig. 8(b–i). To enable the vehicle to accelerate smoothly and rapidly when acceleration is demanded and to decelerate rapidly when necessary, it is requisite to categorize and handle the speed ratio step values of the acceleration and deceleration gears of the AT. This can be accomplished by screening through the density distribution of the points in the speed ratio step graphs corresponding to the acceleration and deceleration gears, and preferentially determining the corresponding speed ratio step value ranges of the acceleration and deceleration gears, as presented in Table 6. From Table 6, it can be seen that for the deceleration gears, the preferred speed ratio step value is within the range of 1.2 to 2.5, which indicates that the speed changes rapidly during the deceleration process, meeting the demand for a quick speed reduction during deceleration. For the acceleration gear, the speed ratio step is close to 1 at ***y***_***5***_, ***y***_***6***_, and ***y***_***7***_, indicating a smooth acceleration process with high comfortability. The speed ratio step is larger at ***y***_***8***_, and the speed increases rapidly, meeting the requirement for fast acceleration in high gears. From the above analysis, it is known that obtaining the optimized value range of the speed ratio step for the acceleration and deceleration gears can guide the structural design of the AT to achieve the optimal performance of the AT.

**Table 6:** Preferred value range for the speed ratio step of each gear.

Figure number	Speed ratio step	Preferred value range
Figure 8b	***y***_***1***_	1.200–1.450
Figure 8c	***y***_***2***_	2.325–2.500
Figure 8d	***y***_***3***_	1.450–1.750
Figure 8e	***y***_***4***_	1.250–1.293
Figure 8f	***y***_***5***_	1.150–1.200
Figure 8g	***y***_***6***_	1.020–1.043
Figure 8h	***y***_***7***_	1.065–1.085
Figure 8i	***y***_***8***_	1.300–1.500

### Optimized transmission data

According to the above discussion, for 91 points in Fig. 8(a), the maximum average efficiency is 0.9572, and the optimal four gear characteristic parameters are *K*_*1*_ = 1.33, *K*_*2*_ = 1.51, *K*_*3*_ = 1.37, and *K*_*4*_ = 3.98 (It is notable that this may not be the ultimate optimal characteristic parameters of the proposed variant CR-CR 9-speed AT. This is merely the optimal solution within this random sampling. To obtain the final optimal values, a larger volume of data sampling is required, which demands greater computing capacity and more time. The purpose of using this optimal value here is to make a comparison with the traditional 9-speed AT, in order to emphasize the superiority of designing the AT using this algorithm). Based on these *K*_*i*_ values, the corresponding transmission ratios and efficiencies for each gear ratio are shown in Table 7. Table 8^27^ shows the transmission ratios and efficiencies for the traditional 9-speed AT. By comparing the data in Tables 7 and 8, it can be seen that the transmission ratio range of the proposed variant CR-CR 9-speed AT is larger. The reason for this result is that some classic 9-speed ATs optimize performance by starting with a speed ratio step, controlling the speed ratio step of each gear in the range of 1.1–1.7 or even smaller, which would result in a smaller transmission ratio range of the AT^26^. However, from Table 7, it can be seen that the speed ratio step of the second and third gear ratios of the proposed variant CR-CR 9-speed AT exceeds 2, reaching 2.339, while the other gear ratios are all in the range of 1 to 1.8. If the speed ratio step in the range of 1.1–1.7 is used as the starting constraint condition according to the traditional method, the optimal transmission scheme may be missed. Furthermore, it can be known from Table 8 that the comprehensive transmission evaluation index (Ct) of the variant CR-CR 9-speed AT designed in this paper is higher than that of other traditional ATs, indicating its good performance. It is worth mentioning that the comprehensive transmission evaluation index is determined by Eq. (17), which is obtained by weighting the transmission ratios (Trs) and transmission efficiencies (Tes) of each gear (The parameters required for calculation can be obtained according to Table 7 in this paper and corresponding Tables in Ref. [14] (page 16)).

17 $$Ct=\sum\limits_{{i=1}}^{n} {\left| {Tr_{1}^{{1.2}}} \right| \times Te_{1}^{{1.5}}}$$

Here, *i* represents the gear number, *Tr* is the transmission ratio of the current gear, *Te* is the transmission efficiency of the current gear, and 1.2 and 1.5 are the corresponding weighting parameters, which are obtained through a comprehensive study of the existing achievements (i.e., Refs. [14–15]) of our team.

As is well known, the transmission ratio is one of the core parameters of the transmission and directly affects the acceleration performance and fuel economy of the vehicle. Through parameter weighting, it can average the extreme influence of the difference in transmission ratios of high and low gears on the performance of the AT. The transmission efficiency reflects the loss situation of energy transfer, and high efficiency means less energy waste. Through parameter weighting, the enhancing effect of high efficiency on performance is further emphasized. The comprehensive transmission evaluation parameter combines the transmission ratio and transmission efficiency organically and can comprehensively reflect the overall performance of the transmission instead of relying solely on a certain index. Compared with the traditional method of directly comparing the average transmission efficiency^26^, it may be more in line with engineering practice and can better reflect the performance in actual use.

**Table 7:** Optimized transmission data for the variant CR-CR 9-speed automatic transmission.

Gear	Transmission ratio (Tr)	Transmission efficiency (Te)
1	6.9755	0.90951
2	5.0589	0.93092
3	2.1626	0.96352
4	1.2512	0.98813
5	1	1
6	0.8636	0.98069
7	0.8402	0.97741
8	0.7839	0.97565
9	0.5909	0.93941
10	− 1.4310	0.90657

**Table 8:** Comparison of parameters between the variant CR-CR 9-speed automatic transmission and the traditional 9-speed automatic transmission.

AT model	Variant CR-CR 9-speed AT	ZF 9HP AT	General motors 9T50E AT	Mercedes-Benz 9G-Tronic AT
Range of transmission ratio	0.5909–6.9755	0.48–4.70	0.62–4.69	0.601–5.503
Comprehensive transmission evaluation index (Ct)	22.90	20.70	22.10	22.09
Speed ratio step				
***y***_***1***_	1.379	1.65	1.42	1.65
***y***_***2***_	2.339	1.50	1.10	1.44
***y***_***3***_	1.728	1.38	1.23	1.39
***y***_***4***_	1.251	1.38	1.28	1.37
***y***_***5***_	1.158	1.25	1.32	1.21
***y***_***6***_	1.027	1.14	1.45	1.16
***y***_***7***_	1.072	1.21	1.33	1.21
***y***_***8***_	1.327	1.21	1.21	1.19

### Functional simulation

**Fig. 9:**
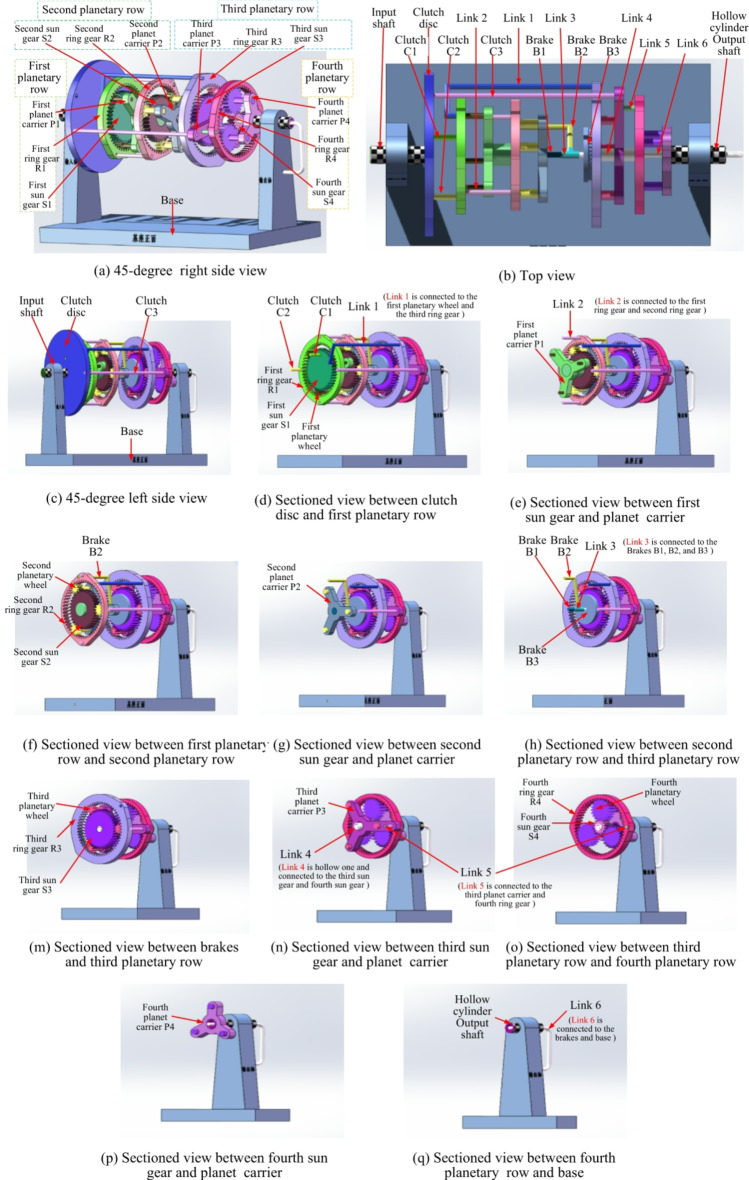

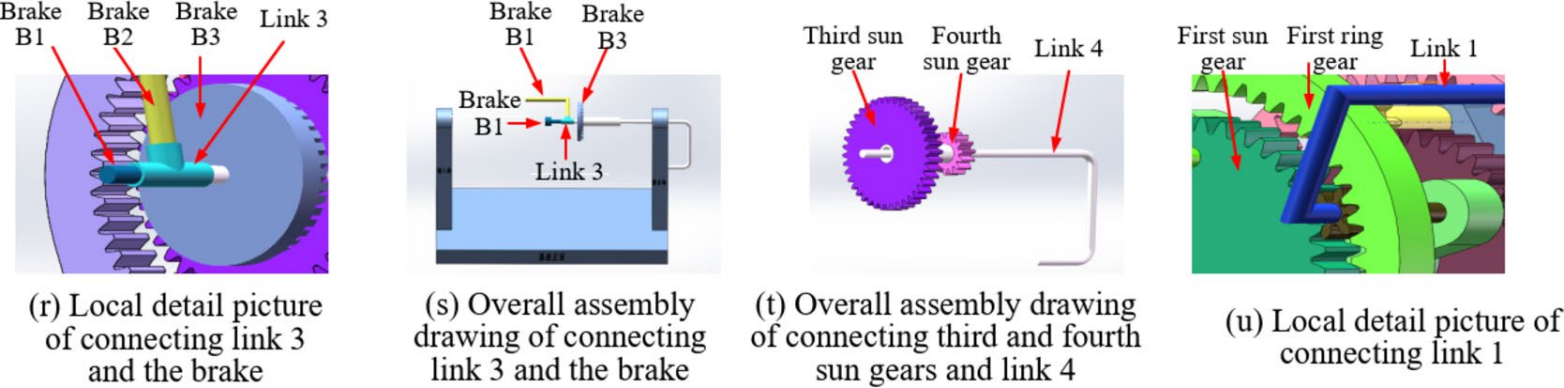
Three-dimensional structural graph of the variant CR-CR 9-speed automatic transmission (the figure was created by the first author Liangyi Nie using SolidWorks 2022 (version: 2022 SP3.1, license number: 00180000 0010 9647 NKHW WBH3, URL link: https://www.solidworks.com/media/solidworks-2022-parts), and the author owns the ownership of the figure).

Based on the determined characteristic parameters of the planetary gear train, the number of teeth and pitch circle information of the respective sun gears, planet gears, and ring gears in the four planetary gear trains can be determined. Generally, the planetary gears of each gear row in the AT adopt standard spur cylindrical gears, with a modulus of 2 and a pressure angle of 20°. From the above characteristic parameters (*K*_*1*_ = 1.33, *K*_*2*_ = 1.51, *K*_*3*_ = 1.37, and *K*_*4*_ = 3.98), it can be known that the parameter information of each gear is shown in Table 9. Utilizing the SolidWorks software, the schematic graph of the proposed variant CR-CR 9-speed AT structure is depicted in Fig. 9 (the figure was created by the first author Liangyi Nie using SolidWorks 2022 (version: 2022 SP3.1, license number: 00180000 0010 9647 NKHW WBH3, URL link: https://www.solidworks.com/media/solidworks-2022-parts)). To facilitate the reading and understanding of readers, Fig. 9 shows the 45-degree right side view, top view, and 45-degree left side view of the assembly in (9a), (9b), and (9c) respectively. Moreover, a series of cross-sectional views of the assembly from left to right are presented, and the corresponding components are labeled respectively, shown in (9d-9q). At the same time, a detailed view of the key connecting links connection is displayed in Fig. 9r and u. Note that the assembly in Fig. 9(b) is positioned differently for easier marking, but this does not affect the presentation result.

**Table 9:** Parameters of sun gears, planet gears, and ring gears.

Planetary designation	Sun gear		Planet gear		Ring gear	
	Number of teeth	Pitch circle	Number of teeth	Pitch circle	Number of teeth	Pitch circle
First planetary row	40	80	6	12	52	104
Second planetary row	36	72	9	18	54	108
Third planetary row	40	80	8	16	56	112
Fourth planetary row	14	28	21	42	56	112

Hereinafter, taking the second gear as an example, the motion simulation process of the variant CR-CR 9-speed AT is introduced. In this gear, clutches C_2_, C_3_, brake B_1_, and the second planetary row do not participate in the movement and will be omitted.

*Clutch operates*: The clutch disc is driven to rotate by the input shaft. The clutch disc is connected to the first sun gear S_1_ through a connecting link, constituting clutch C_1_ and thereby exerting a driving effect.

*Brakes operate*: The connecting link fixed to the base enters from the output shaft, passes through the hollow cylinder connecting the fourth sun gear S_4_ and the third sun gear S_3_, and is fixed to the disc brake installed on the fourth sun gear S_4_. Through friction, the rotation of the third sun gear S_3_ and the fourth sun gear S_4_ is restricted, constituting brake B_3_. Further, this connecting link is connected to the second ring gear R_2_ through a reversing device. Between the second ring gear R_2_ and the first ring gear R_1_, the rotation of the two ring gears is restricted by a fixed connection, constituting brake B_2_, thereby achieving the braking effect.

*Motion process*: The clutch disk drives the first sun gear S_1_ to operate, enabling the rotation of the first planet carrier P_1_, the second sun gear S_2_, and the second planet carrier P_2_. Simultaneously, the first planet carrier P1 drives the third ring gear R_3_ to rotate, causing the third planet carrier P_3_ to drive the fourth ring gear R_4_ and ultimately propelling the fourth planet carrier P_4_, which is connected to the output shaft, to rotate, constituting the motion process of the second gear. It is necessary to clarify that due to the limited conditions of the school and the lack of the desired data acquisition and analysis equipment, it is currently impossible to conduct a prototype instance verification. This portion of the content may be further elaborated in future work.

## Conclusions

This paper proposes a performance analysis and optimization approach for a variant CR-CR 9-speed AT. It can not only enhance the comprehensive performance of the 9-speed transmission but also offer new directions and concepts for the in-depth development of multi-gear ATs, providing technical support and impetus for practical applications. The conclusions and advantages can be summarized as follows:

1. A novel variant of the CR-CR 9-speed AT configuration was proposed, providing assistance in promoting the research and development as well as application of multi-speed ATs.
2. The kinematics and dynamics of this type of 9-speed AT were analyzed by means of the lever method, and the general formula for calculating the speed ratio and efficiency was obtained.
3. Based on the general formula for calculating the speed ratio and efficiency, a scale optimization algorithm was proposed with the level of the final transmission efficiency as the indicator. A three-dimensional model was established through examples for simulation and verification, demonstrating the validity of the algorithm.

## Data Availability

The datasets used and/or analysed during the current study available from the corresponding author on reasonable request.
